# Effects of High Fat Feeding and Diabetes on Regression of Atherosclerosis Induced by Low-Density Lipoprotein Receptor Gene Therapy in LDL Receptor-Deficient Mice

**DOI:** 10.1371/journal.pone.0128996

**Published:** 2015-06-05

**Authors:** Florian Willecke, Chujun Yuan, Kazuhiro Oka, Lawrence Chan, Yunying Hu, Shelley Barnhart, Karin E. Bornfeldt, Ira J. Goldberg, Edward A. Fisher

**Affiliations:** 1 Division of Endocrinology, Diabetes and Metabolism, New York University Langone Medical Center, New York, New York 10016, United States of America; 2 Division of Cardiology, New York University Langone Medical Center, New York, New York 10016, United States of America; 3 Department of Molecular and Cellular Biology, Baylor College of Medicine, Houston, Texas 77030, United States of America; 4 Department of Medicine, Division of Metabolism, Endocrinology and Nutrition, Diabetes and Obesity Center of Excellence, University of Washington, Seattle, Washington 98109, United States of America; University of Milan, ITALY

## Abstract

We tested whether a high fat diet (HFD) containing the inflammatory dietary fatty acid palmitate or insulin deficient diabetes altered the remodeling of atherosclerotic plaques in LDL receptor knockout (*Ldlr^-/-^*) mice. Cholesterol reduction was achieved by using a helper-dependent adenovirus (HDAd) carrying the gene for the low-density lipoprotein receptor (*Ldlr*; HDAd-LDLR). After injection of the HDAd-LDLR, mice consuming either HFD, which led to insulin resistance but not hyperglycemia, or low fat diet (LFD), showed regression compared to baseline. However there was no difference between the two groups in terms of atherosclerotic lesion size, or CD68+ cell and lipid content. Because of the lack of effects of these two diets, we then tested whether viral-mediated cholesterol reduction would lead to defective regression in mice with greater hyperglycemia. In both normoglycemic and streptozotocin (STZ)-treated hyperglycemic mice, HDAd-LDLR significantly reduced plasma cholesterol levels, decreased atherosclerotic lesion size, reduced macrophage area and lipid content, and increased collagen content of plaque in the aortic sinus. However, reductions in anti-inflammatory and ER stress-related genes were less pronounced in STZ-diabetic mice compared to non-diabetic mice. In conclusion, HDAd-mediated *Ldlr* gene therapy is an effective and simple method to induce atherosclerosis regression in *Ldlr^-/-^* mice in different metabolic states.

## Introduction

Patients with both type 1 and type 2 diabetes mellitus (T1DM, T2DM) have an increased risk of cardiovascular disease. The regression of atherosclerotic plaques would be a major advancement in the treatment of this disease. With the large-scale use of statin medications, clinical studies have shown that over 60% of patients with marked reduction of circulating cholesterol concentrations have some reduction in plaque volume [1], but these changes reach at most, a few percent over 1–2 years. Diabetes partially negates this reduction in human atherosclerosis [2]. Defective regression in patients with diabetes may be due to the effects of insulin resistance or hyperglycemia on systemic risk factors, such as lipoprotein levels, blood pressure, and as we recently have shown, leukocytosis [3]. In addition, metabolic effects of diabetes may have direct effects on the arterial wall [4]. Convenient pre-clinical models to study the mechanisms by which diabetes adversely affects atherosclerosis, particularly its regression, would be valuable.

There are several strategies to study atherosclerosis regression in animal models. Our laboratory has generated a surgical mouse model of atherosclerotic regression using a transplantation strategy in *ApoE* ^*-/-*^ mice [5, 6], and in recent studies, has replicated this model in LDLr-/- mice as well (C. Yuan, I. Goldberg, E. Fisher, unpublished). While a valuable model, it contains inherent problems: the transplantation surgery is complicated and only a limited number of animals can be studied. An alternative regression model that we have generated is based on the Reversa mouse [7]. In this model, the hypercholesterolemia of the *Ldlr* ^*-/-*^ mouse is eliminated via conditional deletion of microsomal triglyceride transfer protein (MTTP). Indeed, while T1DM impaired atherosclerosis regression in these mice [8], the model’s general usefulness for T1DM and T2DM studies is limited by its genetic complexity: there are four genetic modifications, making the manipulation of candidate factors by breeding with transgenic or knockout mice extremely time-consuming and expensive. In order to overcome this limitation, we tested whether a gene therapy approach that reintroduces LDL receptors (LDLR) into any genetically altered mouse on the *Ldlr* ^*-/-*^ background could be adapted to the study of acute atherosclerosis regression in diabetes and other metabolic perturbations. Induction of atherosclerosis regression by gene therapy has been achieved by other groups [9–14], but so far no one has used this approach to study atherosclerosis regression in diabetic mice.

Previous results showed that helper-dependent adenovirus (HDAd) carrying the *Ldlr* gene (denoted HDAd-LDLR) reduced circulating cholesterol levels and retarded atherosclerotic lesion progression in *Ldlr* ^*-/-*^ mice [15] and promoted regression 28 weeks after vector injection [14]. The helper-dependent adenovirus (also known as gutless adenovirus) used for this study is the latest generation adenovirus that is devoid of all adenoviral protein genes. It enables easy and safe administration with high transduction efficiency *in vivo* [16–19], making it a useful tool for generation of a non-surgical atherosclerotic regression model. Whether such a strategy would show similar effects in diabetic *Ldlr* ^*-/-*^ or *Ldlr* ^*-/-*^ mice fed a high fat diet (HFD), frequently used to make mouse models of the metabolic syndrome, is untested.

In the present report, therefore, we used helper-dependent adenoviral expression of the LDLR to determine if regression was adversely affected by either an incipient metabolic syndrome-like phenotype by feeding a palmitate-containing HFD or a T1DM-like phenotype created using streptozotocin (STZ)-induced insulin deficiency. Changes in atherosclerosis at different sites were assessed (plaque composition, size, and macrophage gene expression). Dramatic beneficial changes in plaque characteristics were observed in both settings, establishing the flexibility of using this approach.

## Materials and Methods

### Animals and their treatments

Male *Ldlr^-/-^* mice on the C57BL/6 background (Jackson Laboratories, Bar Harbor, ME) were used. All procedures were approved by the Animal Care and Use Committee at New York University Langone Medical Center and Columbia University Medical Center. Mice were maintained in a temperature-controlled (25°C) facility with a 12-h light/dark cycle and given free access to water and food, except when fasting blood specimens were obtained. Mice were fed a laboratory rodent diet as indicated. Euthanasia at completion of study was achieved by intraperitoneal injection of ketamine100mg/kg and xylazine 10mg/kg followed by cervical dislocation. A total number of 120 mice were used.

To study the effect of aggressive lipid lowering on atherosclerosis regression in a model of incipient metabolic syndrome, *Ldlr* ^*-/-*^ mice were first fed a high cholesterol diet (HCD, Research Diets, D01061401C, 10 kcal% fat, 0.15% cholesterol) for 16 weeks to develop complex atherosclerotic plaques, as described [20]. A group of mice (baseline) was sacrificed after 16 weeks to assess baseline atherosclerosis, plasma lipid and glucose levels. The remaining mice then received a matched diet with no added cholesterol (D12102C, 10 kcal% fat, 0% cholesterol). Both diets were matched for carbohydrate, protein and fat content. Since switching to a 0% cholesterol diet did not normalize plasma cholesterol levels after 4 weeks, we additionally treated the *Ldlr* ^*-/-*^ mice with the HDAd-LDLR described in [14]. Mice were then divided into one group that received a HFD (Research Diets D13052906, 60 kcal% fat, 0% cholesterol) and a second group that received a matched low fat diet (LFD) (Research Diets D13052902, 10 kcal% fat, 0% cholesterol) (see S1 Table for specific diet contents). The HFD was enriched in palm oil (60% of the calories). Both HFD and LFD were depleted of cholesterol to achieve maximal cholesterol reduction. Palm oil in the HFD was replaced by corn starch in the LFD. After 2 weeks on respective diets, all mice were sacrificed.

To study the effect of HDAd-LDLR-induced regression in normal and diabetic mice, *Ldlr* ^-/-^ mice were weaned at 1 month onto a Western diet (WD) (Research Diets catalog No.D01022601, 41 kcal% fat, 0.15% cholesterol), which was continued for 16 weeks to develop complex atherosclerotic plaques. After 16 weeks a group of mice (baseline) was sacrificed, with plasma and tissue collected as described below. To reduce plasma cholesterol levels and induce atherosclerotic plaque regression, HDAd-LDLR was given after 16 weeks on WD. Mice were injected intravenously (IV) with one dose of either the control empty adenovirus (HDAd-0, 1.0 x 10^11^ viral particles in 100 μl 1xPBS per mouse) or the HDAd-LDLR; the viruses were produced by Gene Vector Core at Baylor College of Medicine. Thereafter, the diet was switched to chow and mice were maintained on this diet for 5 weeks before sacrifice.

To induce insulin-deficient diabetes, mice were injected intraperitoneally (IP) with low dose STZ (Sigma-Aldrich, MO) (7.5mg/kg for five consecutive days) or vehicle at 14 weeks of WD according to the Animal Models of Diabetic Complications Consortium (AMDCC) protocol [21] and as described previously [8, 22]. Two weeks later, at the beginning of the hyperglycemic period, a group of STZ-diabetic mice (baseline, at 16 weeks of WD) was sacrificed and plasma and tissue collected for further analysis as described below. Similar to the non-diabetic mice, the remaining STZ-diabetic mice were then treated with either the HDAd-0 or the HDAd-LDLR at 16 weeks and maintained on chow diet for 5 weeks.

Both the normoglycemic and hyperglycemic mice were sacrificed 5 weeks after the adenovirus injections, after fasting for 6 h. Mice were anesthetized first, then blood, aortic roots, aortic arches, and livers were collected. For each mouse, an aliquot of blood was used for glucose determination, and the remaining blood was immediately centrifuged at 10,000 rpm for 5 min, and the supernatant was used for measuring total cholesterol and triglyceride levels, as well as the cholesterol contents of fast performance liquid chromatography fractions. Aortic roots and arches, including the brachiocephalic artery (BCA), were collected after perfusion with a 10% sucrose/PBS solution, and were then embedded in Tissue Tek OCT and frozen and stored at -80°C.

### Biochemical measurements

#### Plasma glucose

The glucometer and strips for measuring blood glucose level were from Bayer Co. (Glucometer Elite XL model). Briefly, a mouse was first fasted for 6 h, then a distal part of the tail was cut and a drop of blood was released and spotted onto the strip.

#### Plasma cholesterol and triglyceride

Reagents for measuring plasma total cholesterol and triglyceride levels were from Wako Chemicals, USA, following the manufacturer’s instructions.

#### Fast performance liquid chromatography

To analyze the lipid/lipoprotein profile from plasma samples in detail, fast performance liquid chromatography (FPLC) was performed on pooled plasma samples from mice within the same group followed by a colorimetric assay for total cholesterol (Wako Chemicals) from each fraction.

#### Lipoprotein subfractions

VLDL (d < 1.006 g/ml), IDL+LDL (d 1.006–1.063 g/ml), and HDL (d 1.063–1.21 g/ml), were separated by sequential density ultracentrifugation of mouse plasma in a TLA 100 rotor (Beckmann Instruments, Palo Alto, CA). Free fatty acids (FFA) were measured using the Wako HR series kit NEFA.

### Histology

#### Histological and morphometric analyses of atherosclerotic plaque

Serial sections (8 μm thick) were obtained by cryosectioning of frozen aortic roots and arches that were embedded in OCT. The sections were then stained either with anti-CD68 antibody to determine macrophage content, Sirius Red for collagen, or Oil Red O for lipid content, as described below.

#### Immunostaining of macrophages

Tissue sections were first fixed in ice-cold acetone for 10 min. After washing with PBS, they were incubated in a solution of 4% rabbit serum for 10 min to block non-specific binding of the CD68 antibody, and then incubated with 1:200 dilution of primary antibody (mouse anti-CD68 antibody; Serotec, NC) for 1 h. Sections were rinsed 3 times in PBS and incubated for 10 minutes with a biotinylated rat anti-mouse secondary antibody. Tissue was rinsed 3X in PBS and then incubated for 5 min in an alkaline phosphatase-conjugated streptavidin solution. Finally, staining was developed using the Vector Red Kit (Vector Laboratories). Staining was stopped by dipping the slides into distilled water. Slides were then dehydrated by sequential immersion into 70%, 80%, 90%, and 100% ethanol, stained with hemaetoxylin for 1 min, and then cleared with xylene for 3 min and mounted in a resinous medium. These CD68+ stained sections were later used as “guide slides” during laser capture microdissection (LCM) in order to assure that all laser-captured material was specific for CD68+ cells (which were mainly monocyte-derived macrophages and foam cells).

#### Sirius red staining

Briefly, tissue section slides were first stained with hemaetoxylin for 8 min, incubated in a picro-sirius red solution for 1 h, then washed 2X in acidified water for 30 min each. Excess water was removed from the sections by vigorous shaking of the slides. Sections were sequentially dehydrated in 70%, 80%, 90%, and 100% ethanol. Finally, slides were cleared in xylene and mounted in Permount. Prepared slides were analyzed using a polarized light microscope to detect collagen content.

#### Oil Red O staining

Oil Red O staining solution was freshly prepared. To stain neutral lipids, tissue section slides were fixed in 10% formalin for 10 min, then washed 2X with water and dipped into 60% isopropanol for 30 s. Slides were then stained with Oil Red O solution for 18 min, dipped into 60% isopropanol for 30 s, washed 2X with water, stained with hemaetoxylin for 5 s, dipped into bluing solution for 1 min, washed again with water, and finally mounted with mounting medium (Vector Laboratories Inc.).

#### En face Oil Red O staining

At the time of sacrifice, the aortic arch and distributing vessels were photographed. The aortas were removed, cut open, fixed in 10% buffered formalin, and stained with Oil Red O. The en face area of the aortic arch and descending aorta, stained by Oil Red O, was quantified relative to its surface area using ImagePro Plus software.

#### Staining & analysis of Brachiocepahlic Arteries (BCA)

BCAs embedded in OCT were serially sectioned (6 μm sections). Every 6^th^ cross section was stained using the Movat’s pentachrome staining method to visualize atherosclerotic lesions. The maximal lesion site was determined, and adjacent sections were immunostained using a Mac2 antibody to detect lesion macrophages, as described previously [23]. Lesion morphology, including early fatty streak-type lesion, presence of thin fibrous cap, acellular core, lesion glycosaminoglycans, lesion collagen, intraplaque hemorrhage, cholesterol clefts, chondrocyte-like cells, and calcification were evaluated in a binary fashion for each section by an observer blinded to the treatment groups, according to a previously described protocol [24]. The frequency of each morphological feature was calculated for each mouse, and then averaged for each group of mice. Presence of medial glycosaminoglycans and collagen were evaluated in a similar manner [24].

#### Morphometric measurements

Morphometric measurements were performed on digitized images of stained serial sections by using IMAGEPRO PLUS software. At least 5 sections per vessel were analyzed and the mean value used as the summary parameter.

### Molecular analyses of atherosclerotic plaques

#### Laser capture microdissection (LCM)

To isolate foam cells from plaques, LCM was performed with the PixCell IIe instrument (Arcturus Bioscience, Mountain View, CA). Briefly, 8 μm frozen sections were dehydrated in ethanol, dipped twice in xylene, and air dried. CD68+ stained slides were was used as guides to identify the locations of macrophages in the serial sections, which were then laser-captured using established procedures in our laboratory [25]. RNA was then extracted and purified from the laser-captured samples by the Qiagen RNeasy MicroIsolation kit, and treated with DNase. The RNA samples were then analyzed for quality and quantity using an Agilent 6100 Bioanalyzer before conversion to cDNA and amplification using a Nugen kit (Nugen Co. USA). Gene expression regulation was subsequently analyzed using quantitative real-time PCR (qPCR).

#### qPCR

Amplified cDNAs (1 ng/sample) from laser-captured macrophages were used for measurement of gene expression by qPCR (Applied Biosystems 7300 real time PCR machine), using a kit from Biorad. The sequences for the primers are given in S2 Table. All data were normalized to cyclophilin A and expressed as fold change over the control group results. For the laser-captured cell data, the results are from 3 independent samples, each representing amplified cDNA originating from the macrophages from one animal.

### Statistical analysis

Data are expressed as mean ± SEM and were analyzed by two-tailed *t* test. For multiple comparisons, data were analyzed by one-way ANOVA followed by Newman-Keuls comparison test. *P* < 0.05 was considered to be significant. All tests were performed using the Prism software (GraphPad Software, Inc., La Jolla, CA).

## Results

### Assessment of short time HFD versus LFD in HDAd-LDLR treated mice

**Fig 1:**
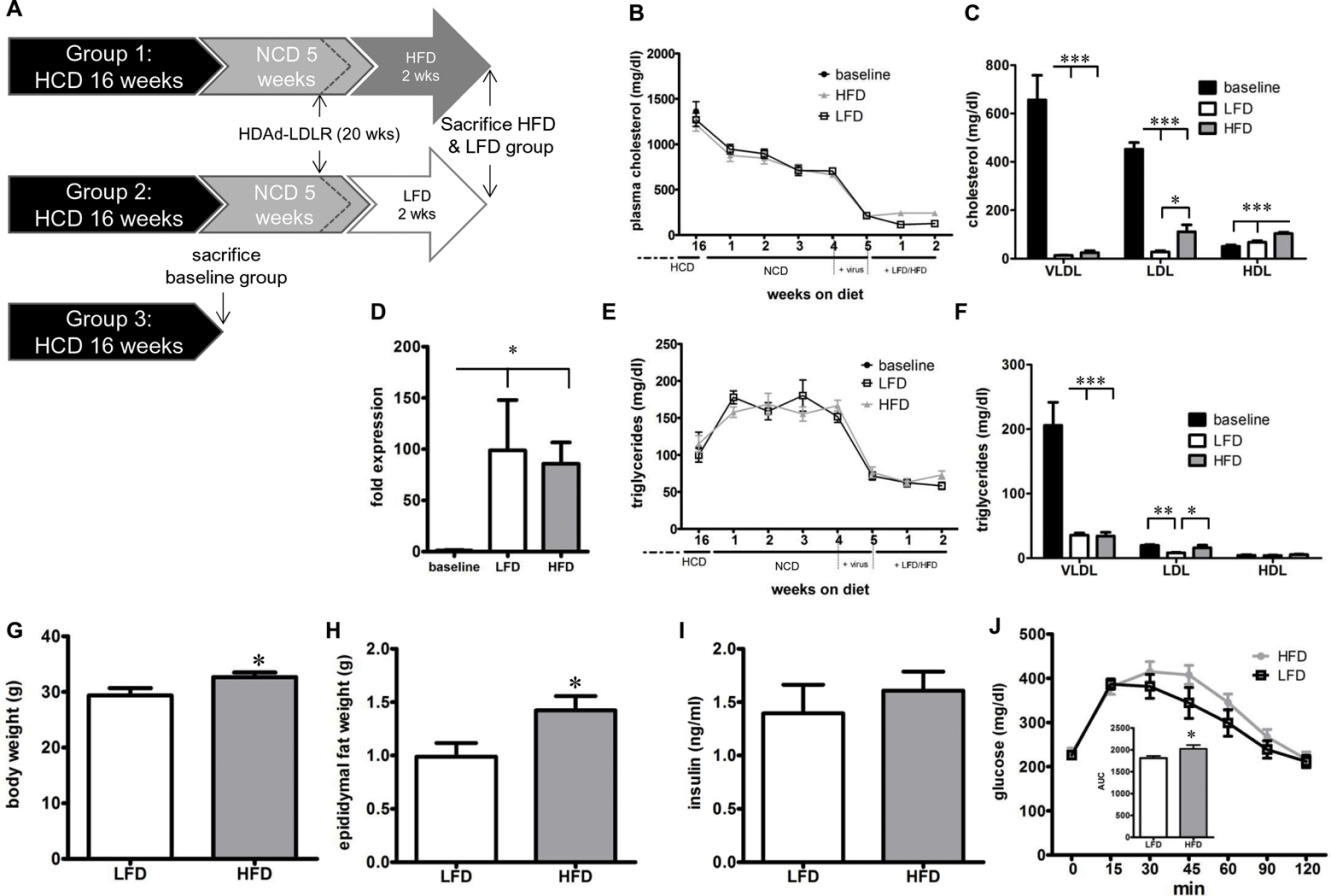
Lipid profile and metabolic phenotype in HFD vs. LFD-fed mice with HDAd-LDLR retreatment. **(A)** Schematic diagram showing the experimental course. Following 16 weeks of HCD, baseline mice were sacrificed. The remaining mice were put on a diet with no added cholesterol (NCD) and treated with the HDAd-LDLR at week 21. At week 22 mice were divided into a HFD and LFD group and sacrificed after 2 weeks. **(B)** Total plasma cholesterol and **(C)** cholesterol subfractions of baseline, LFD and HFD-fed mice. **(D)** Hepatic LDLR mRNA expression before and after (HFD & LFD) HDAd-LDLR treatment. **(E)** Total plasma triglycerides and **(F)** triglyceride subfractions of baseline, LFD and HFD-fed mice. **(G)** Body weight, **(H)** epididymal fat weight, **(I)** plasma insulin levels and **(J)** glucose tolerance test, inset: AUC for glucose tolerance test. Baseline n = 11, LFD = 10, HFD = 9. * p < 0.05, ** p < 0.001, *** p < 0.001.

To study the effects of a HFD containing the inflammatory dietary fatty acid palmitate on atherosclerosis regression, mice were fed a HCD (0.15% cholesterol, with 10 kcal% fat, 70 kcal% carbohydrate and 20 kcal% protein) for 16 weeks, followed by a matched diet with no cholesterol (NCD) (Fig 1A). We selected this HCD with relatively low fat content to avoid the development of a metabolic syndrome before the induction of atherosclerosis regression and because a higher fat content would have increased the cholesterol content in the otherwise cholesterol-free diet after induction of regression.

A baseline group was sacrificed after 16 weeks of HCD. Plasma cholesterol and triglyceride levels in the baseline group were 1362 ± 108 mg/dl and 111 ± 20 mg/dl, respectively. The majority of cholesterol was confined to the VLDL and LDL fractions, whereas triglycerides were mostly in VLDL particles (Fig 1C & 1F). As the NCD failed to reduce plasma cholesterol to levels required for regression (684±18 mg/dl after 4 weeks) we treated these mice with a single IV injection of the HDAd-LDLR virus to restore hepatic LDL receptor expression (Fig 1D). Within a week, plasma cholesterol levels decreased to 200±10 mg/dl (Fig 1B). These mice were then divided into two groups, one group receiving a HFD and a second group receiving matched LFD (see S1 Table for specific diet content). There was no cholesterol in either diet. We chose palm oil as the main fat source since lard, the main fat component of 60% HFD commonly used for diet-induced obesity, contains cholesterol. Plasma cholesterol stayed at low levels for the remaining 2 weeks, however mice in the HFD group had slightly higher cholesterol levels (229±29 mg/dl) compared to the LFD group (126±10 mg/dl) (Fig 1B) due to both higher LDL and HDL-cholesterol subfractions (Fig 1C). Plasma triglyceride levels also decreased with HDAd-LDLR treatment and those mice fed HFD had slightly higher levels than mice fed LFD (Fig 1E & 1F). After 2 weeks on HFD, body weight and epididymal adipose tissue had increased significantly in the HFD group (29.4 ± 1.3g in LFD versus 32.7 ± 0.8g in HFD, p<0.05 and 0.988 ± 0.129 mg vs. 1.422 ± 0.135mg, p<0.05, respectively) (Fig 1G & 1H). Glucose tolerance was modestly impaired in the HFD mice; the area under the curve for the glucose tolerance test was significantly increased by 12% (p < 0.05) without change in insulin levels (Fig 1I & 1J). Thus, although not diabetic, these mice had developed insulin resistance and greater adiposity, which we interpreted as incipient metabolic syndrome.

### HDAd-LDLR treatment changed plaque composition in both HFD and LFD mice

**Fig 2:**
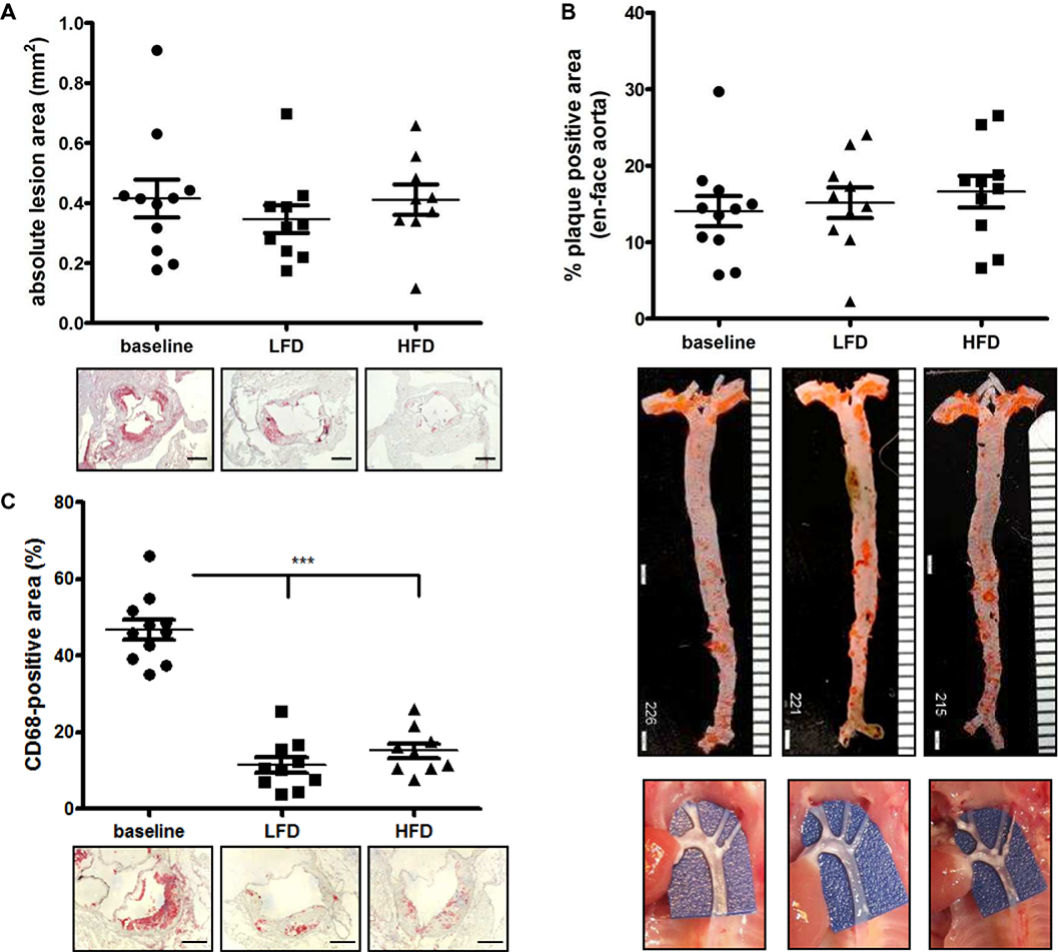
Atherosclerosis regression in mice fed a HFD versus LFD (I). **(A)** Quantification of the absolute lesion area in baseline, LFD and HFD-fed mice of the aortic roots with representative pictures for each group. Bar = 400μm **(B)** Quantification of plaque positive area of en face aortas, including representative picture of en face aortas and in-site pictures (below). **(C)** Quantification of CD68+ area in the aortic sinus. Representative pictures are shown below. Baseline n = 11, LFD = 10, HFD = 9. *** p < 0.001.

**Fig 3:**
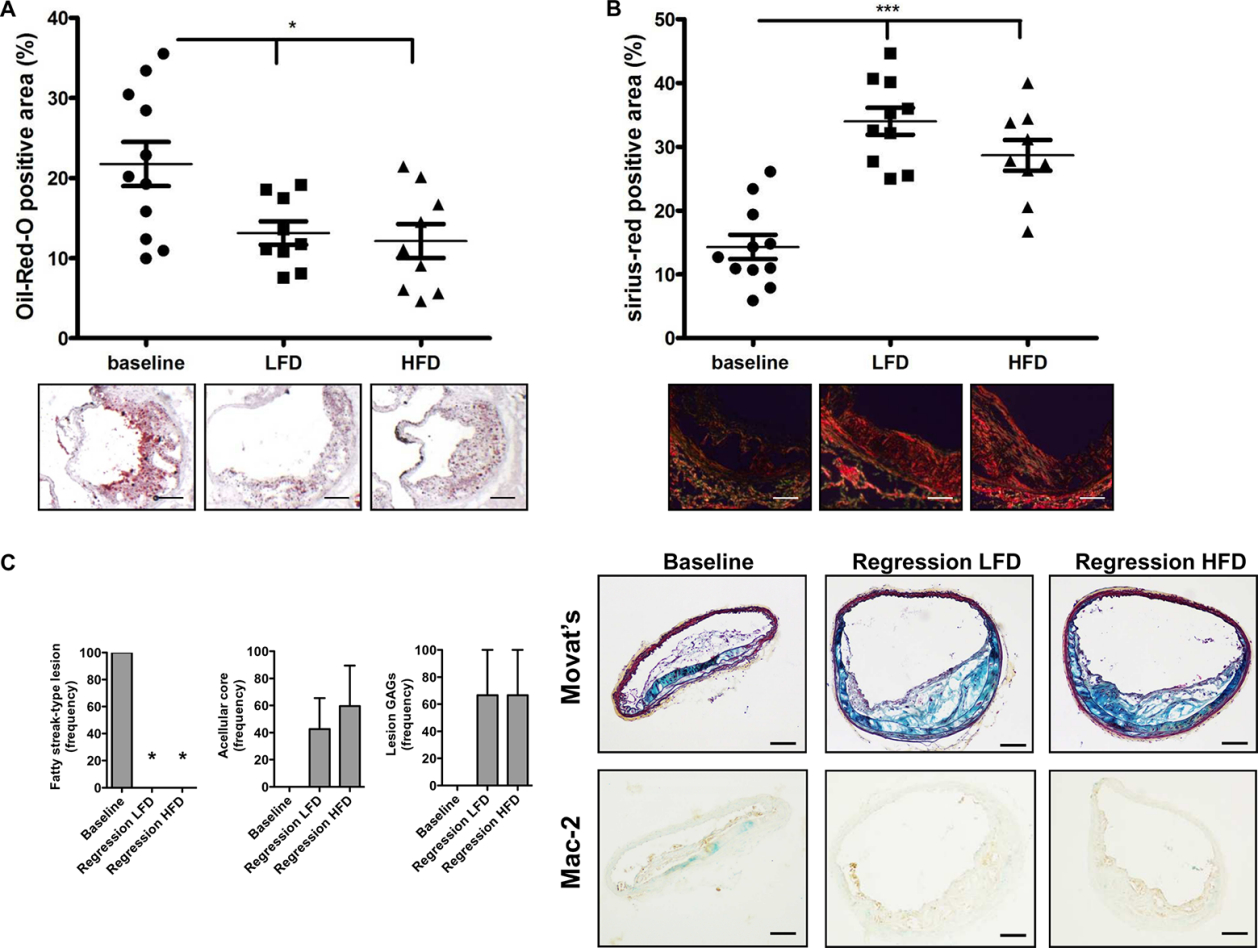
Atherosclerosis regression in mice fed a HFD versus LFD (II). **(A)** Quantification of the Oil-Red-O positive area in the aortic sinus of baseline, LFD and HFD-fed mice. Representative pictures for each group are shown below. Bar = 200μm. **(B)** Quantification of sirius-red positive area of the aortic sinus, including representative pictures of en face aortas and in-site pictures (below). Bar = 200μm. Baseline n = 11, LFD = 10, HFD = 9. * p < 0.05, *** p < 0.001. **(C)** Assessment of atherosclerosis regression in brachiocephalic arteries. Representative pictures of Movat’s staining and Mac-2 staining are shown. n = 3/group. Bar = 100 μm

To study if palmitate-containing HFD or LFD had an effect on atherosclerosis regression promoted by HDAd-LDLR treatment, we studied parameters of atherosclerosis in the aortic root, en face aorta, and BCA. Despite higher plasma cholesterol, triglycerides and early signs of a metabolic syndrome in the HFD mice, CD68-staining, Oil Red O and collagen contents were not significantly different between the two regression groups. Surprisingly, lesion size in the aortic root and in the en face aorta were comparable among the groups and failed to show regression from baseline (Fig 2A and 2B). In contrast, both regression groups showed a dramatic decrease of CD68+ macrophages in the sinus (47±9% CD68+ area in the aortic sinus in baseline group vs. 11±7% in LFD and 15±6% in HFD, Fig 2C). The aortic root lesion Oil Red O positive area was also decreased in both the HFD and LFD groups (Fig 3A). Collagen content in the aortic root, an indicator of vascular remodeling, increased in both regression groups (Fig 3B).

We also assessed lesion regression in the BCA. The baseline group exhibited small fatty streak-like lesions without the presence of acellular cores, lesion glycosaminoglycans or features of more advanced lesions. Both HFD and LFD mice had a high content of lesional glucosaminoglycans and acellular cores compared to baseline (Fig 3C). As noted in the aortic root, we found no difference in regression in BCAs obtained from a subset of mice on each diet. None of the groups exhibited lesions characterized by intraplaque hemorrhage, presence of chondrocyte-like cells or calcification (Fig 3C). The baseline group appeared to have more macrophages/lesion area, but no differences were observed between the HFD and LFD groups (Fig 3C).

### HDAd-LDLR treatment reduced circulating cholesterol in normoglycemic *Ldlr* ^*-/-*^ mice

Because of the efficacy of HDAd-LDLR treatment in the glucose-intolerant, but normoglycemic, HFD mice, we then tested whether higher levels of hyperglycemia would lead to defective regression using the same HDAd-LDLR-viral treatment.

**Fig 4:**
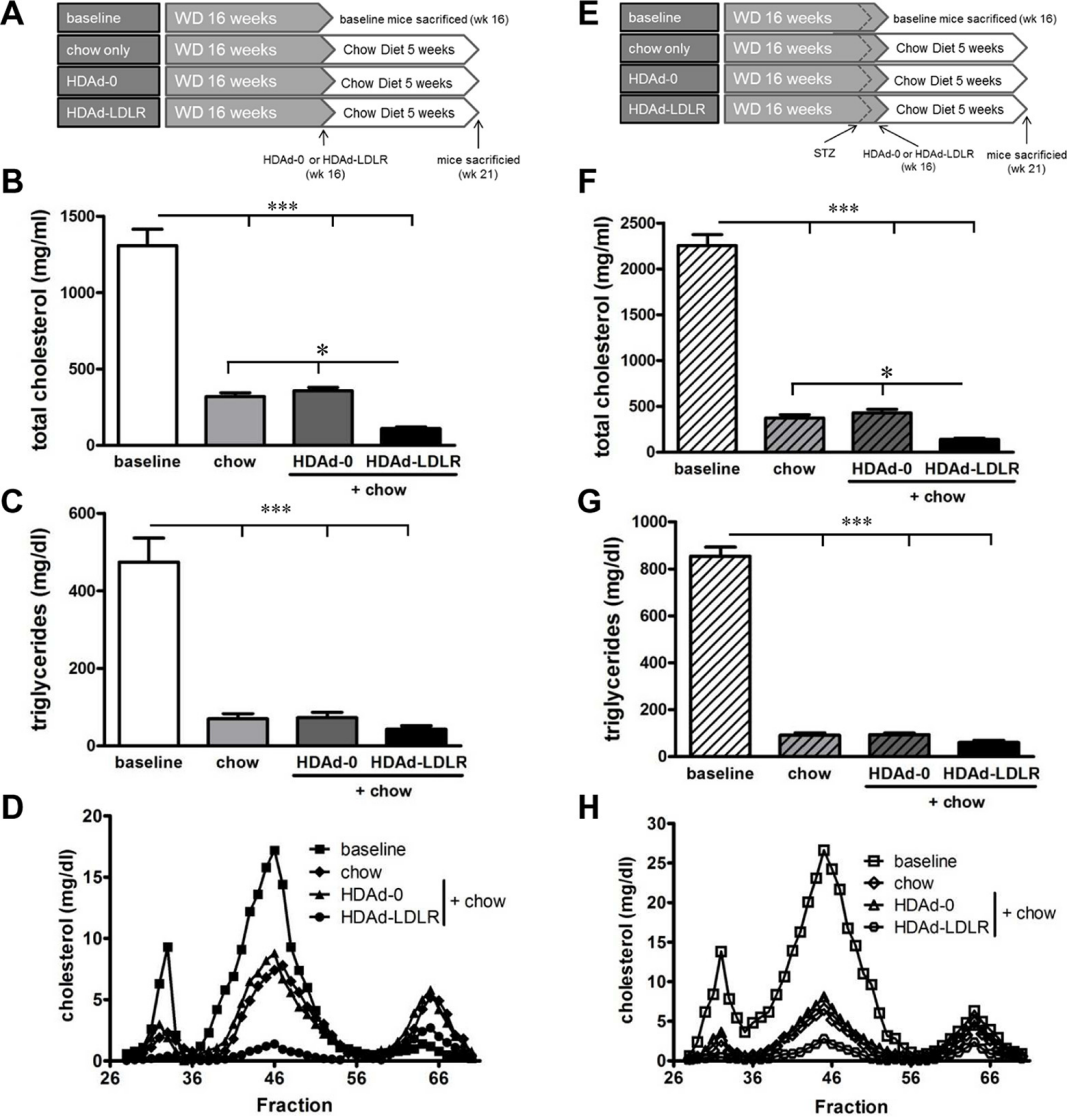
Lipid profiles in non-diabetic and STZ-diabetic *Ldlr* ^*-/-*^ mice using the HDAd-LDLR. Schematic diagram showing the experimental course for the non-diabetic **(A)** and STZ-diabetic **(E)** *Ldlr* ^*-/-*^ mice. Plasma lipids of non-diabetic mice are shown in the left column and of STZ-diabetic mice in the right column. **(B & F)** Respective plasma total cholesterol and **(C & G)** plasma triglyceride levels after various treatments. **(D & G)** FPLC analysis of plasma cholesterol levels after various treatments. N = 10/group, * p < 0.05, *** p < 0.001.

Prior to this, we first assessed the effects of the HDAd-LDLR on circulating lipid levels. *Ldlr* ^*-/-*^ mice were fed with WD for 16 weeks and had baseline cholesterol levels of 1,307±107 mg/dl (Fig 4B). Mice were then either sacrificed (baseline group) or switched to chow diet for 5 weeks (regression groups). After the diet switch, these regression mice were further divided into three groups: 1) no additional treatment (chow diet only, control 1), 2) treatment with HDAd-0 (control 2), or 3) treatment with HDAd-LDLR (Fig 4A). Treatment with HDAd-0 or chow diet only decreased plasma cholesterol levels to 357±22 mg/dl and 320±25 mg/dl, respectively (p<0.001). IV Injection with HDAd-LDLR and concomitant switch to chow diet for 5 weeks led to a further decrease of plasma total cholesterol levels (110±11 mg/dl, p <0.05 vs. chow only and HDAd-0). Plasma triglycerides were significantly reduced in mice on chow diet only, and with additional HDAd-0 or HDAd-LDLR treatment (Fig 4C). Plasma lipoprotein profiles studied by FPLC analysis are shown in Fig 4D. Baseline mice had a small VLDL cholesterol (VLDL-C) peak, a prominent LDL-C peak and a minor HDL-C peak. In the two control groups, the VLDL-C and LDL-C peaks were markedly reduced compared to the baselines. Treatment with the HDAd-LDLR further reduced both VLDL-C and LDL-C peaks, with little change in HDL.

### HDAd-LDLR treatment reduces hypercholesterolemia in diabetic *Ldlr* ^*-/-*^ mice

Mice made diabetic by IP injections of STZ after 14 weeks on WD had glucose levels that increased rapidly, reaching a plateau of around 400 mg/dl within 2 weeks. STZ-diabetic mice were then switched to chow diet and treated with either chow diet only, the empty control virus HDAd-0 or the HDAd-LDLR (Fig 4E). STZ-diabetes increased plasma total cholesterol levels at baseline compared to normoglycemic baseline mice (2,254 ±119 mg/dl) (Fig 4F). Similar to the normoglycemic groups, treatment with either switch to chow diet only or HDAd-0 reduced total plasma cholesterol levels to 374±36 mg/dl and 429±38 mg/dl, respectively. Treatment with HDAd-LDLR further reduced plasma cholesterol in the STZ-diabetic mice (142±18 mg/dl compared to chow only and to HDAd-0 (p <0.05). These were similar levels to those in non-diabetic, HDAd-LDLR-treated animals (120±13 mg/dl). Plasma triglycerides followed a similar pattern as seen in treated non-diabetic mice (Fig 4G). FPLC profiles in the baseline STZ-diabetic mice displayed a similar pattern compared to non-diabetic mice, but with higher cholesterol contents in all three lipoprotein fractions (Fig 4H). Overall, treatment with the HDAd-LDLR led to a marked reduction in non-HDL lipoproteins, compared with baseline, chow diet only and HDAd-0.

### HDAd-LDLR treatment decreases aortic root plaque size and macrophage content

**Fig 5:**
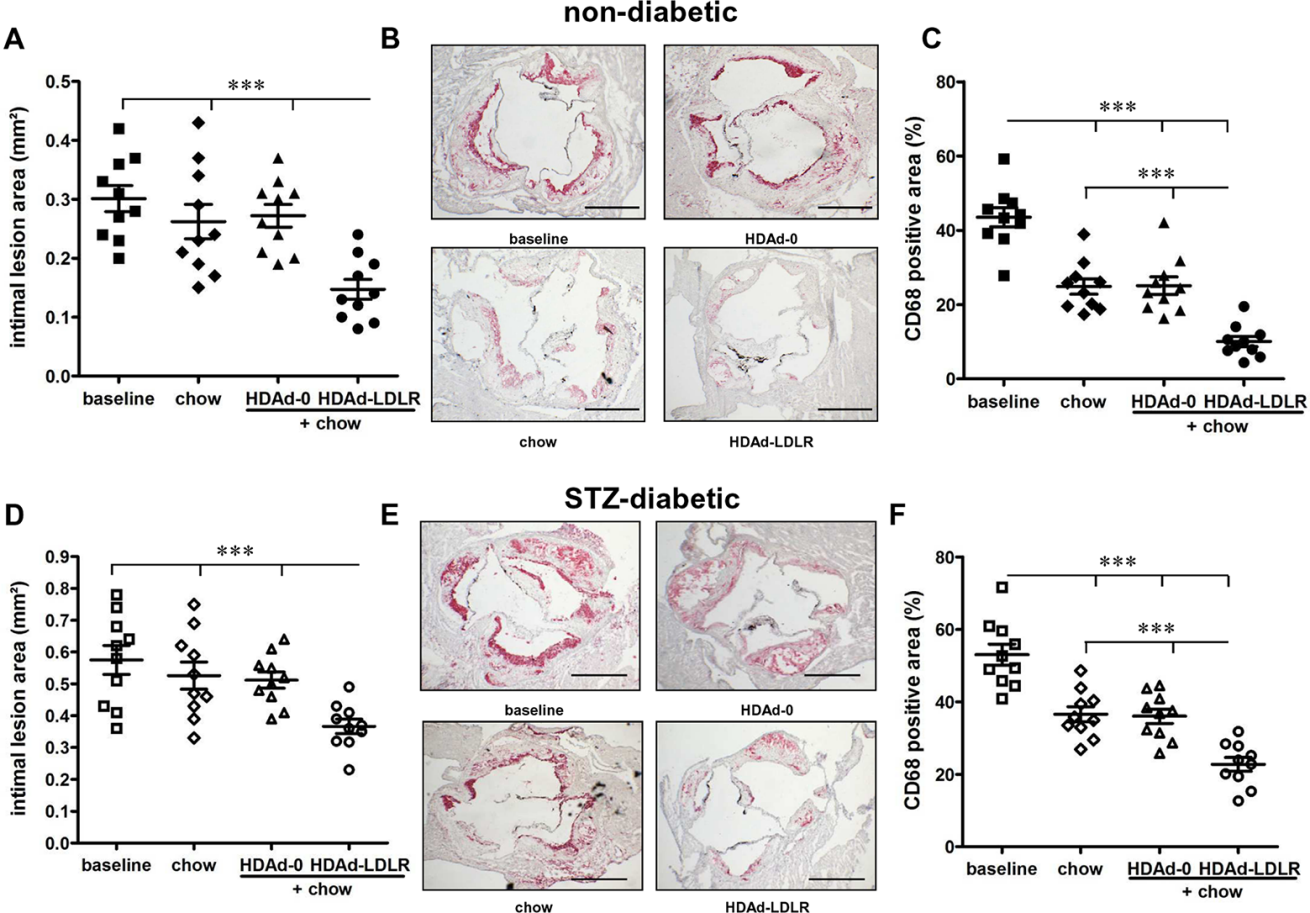
Atherosclerosis Regression in non-diabetic and STZ-diabetic *Ldlr-* ^*/-*^ mice using the HDAd-LDLR. Non-diabetic mice: **(A)** lesional area in the aortic sinus. **(B)** Representative pictures of CD68+ staining of aortic sinus (bar = 500μm) and **(C)** analysis of CD68+ areas of the aortic sinus. STZ-diabetic mice: **(D)** lesional area in the aortic sinus **(E)** Representative pictures of CD68-staining of aortic sinus (bar = 500μm) and **(F)** analysis of CD68+ areas of the aortic sinus. N = 10/group, *** p < 0.001.

We next quantified changes in lesion area at the aortic sinus and plaque macrophage content with lipid reduction in non-diabetic mice. We did not study other sites as our previous study had shown them to be less sensitive indicators or regression. As shown in Fig 5A, the average baseline lesion area at the aortic root was 0.301±0.022 mm^2^ (n = 10). Plaque size in both chow diet only and HDAd-0 + chow diet groups did not differ compared to baseline. Only treatment with HDAd-LDLR reduced aortic sinus plaque area by approximately 50% to 0.147±0.017 mm^2^ (p<0.001 compared with baseline).

CD68+ content as a percent of plaque area was 44±3% in the baseline mice (Fig 5B & 5C). In contrast to the atherosclerotic lesion size, CD68+ plaque macrophage content was reduced to ~25% in both control groups (chow diet only and HDAd-0 + chow diet; a 43% reduction compared with baseline). Additional HDAd-LDLR treatment further decreased CD68+ macrophage content to 10±1% (p<0.001, a 75% reduction compared with baseline). Accordingly, lipid content was decreased by 51% and collagen content increased by 75% in HDAd-LDLR treated mice, changes that are thought to represent a more stable human plaque phenotype (S1 Fig) [26]. In contrast, lipid content in the two control groups decreased by only 25%, whereas collagen content did not change significantly compared to baseline. These results indicate that diet change alone is sufficient to reduce lesion macrophage content, though greater reductions are seen with the HDAd-LDLR treatment. In addition, a more dramatic cholesterol reduction appears to be required to increase lesion collagen and to reduce lesion size.

### Atherosclerotic lesions are greater in STZ-diabetic mice, but also regress

Baseline STZ-diabetic mice displayed markedly increased lesion area (0.57±0.045 mm^2^, n = 10) and CD68+ plaque area (53±3%) compared to non-diabetic mice (Fig 5D–5F). Restoration of the LDLR receptor by HDAd-LDLR treatment significantly decreased aortic root lesion size (0.368±0.022 mm^2^, a 36% decrease versus STZ baseline), whereas lesion size was not affected in the two control groups (chow only and HDAd-0 + chow) (Fig 5D). Similar to the non-diabetic mice, there was a further decrease in CD68+ macrophages with HDAd-LDLR treatment (23±2%), a reduction by 57% compared to the STZ baseline group. Lipid content decreased and collagen content increased in these mice by 49% and 75%, respectively (S2 Fig). Lipid content also decreased in the two control groups, albeit only 16–23%. Collagen content did not differ significantly in the control groups compared to baseline. Comparing the percentage reduction of non-diabetic versus STZ-diabetic HDAd-LDLR treated mice revealed a significant impairment of the regression of CD68+ cells in STZ-diabetic mice; in non-diabetic mice CD68+ cells decreased by 77% versus a 57% decrease in STZ-diabetic mice (p<0.01) The percentage difference in plaque size regression between non-diabetic and STZ-diabetic mice also showed a trend to decrease less in the latter (36% vs. 51%, respectively).

### Changes in macrophage gene expression after HDAd-LDLR treatment

**Fig 6:**
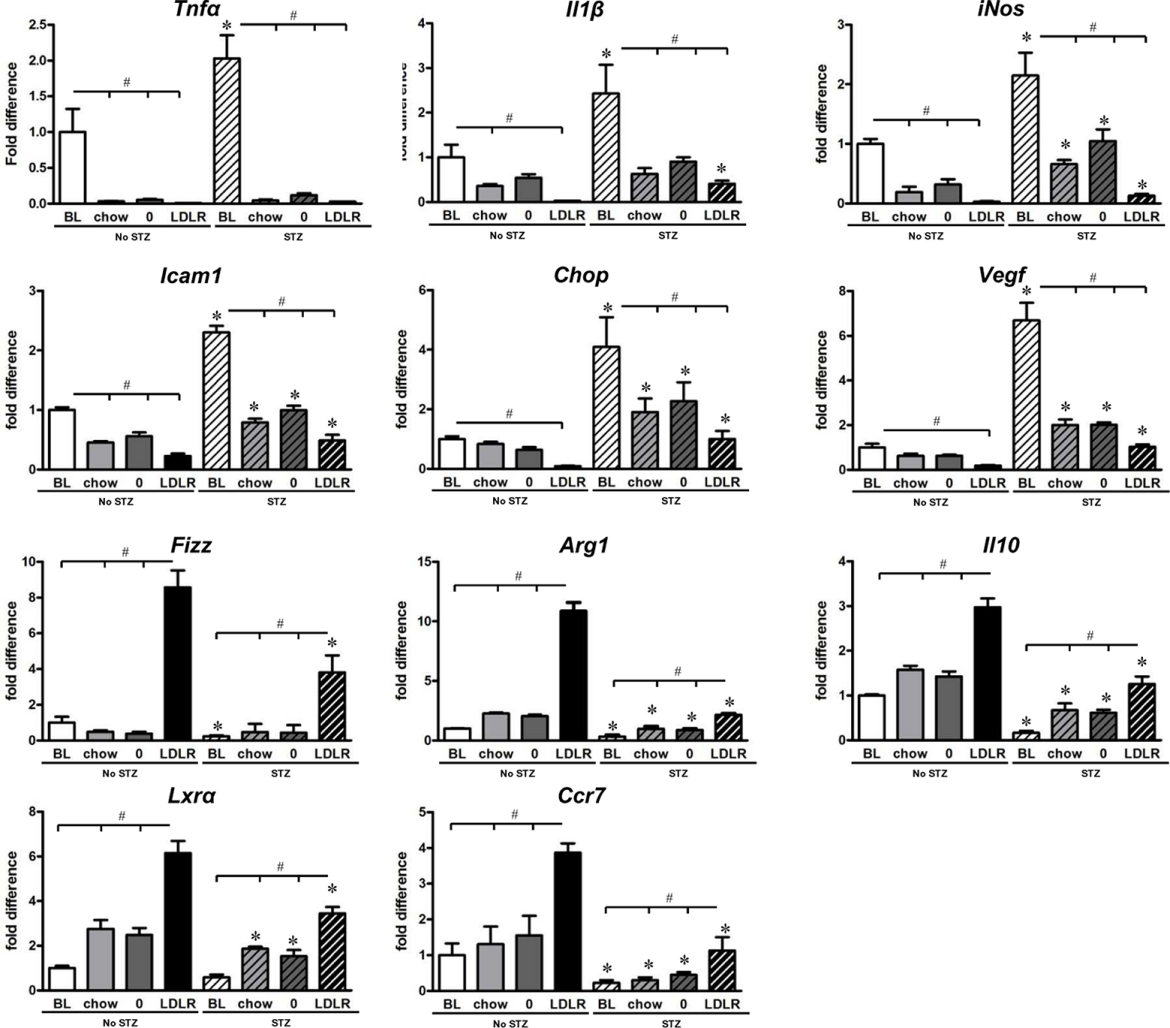
Gene expression analysis of laser-captured plaque CD68+ cells of non-diabetic *Ldlr* ^*-/-*^ and STZ-diabetic *Ldlr* ^*-/-*^ mice. CD68+ cells from aortic roots were isolated using LCM. mRNA was then isolated and subjected to analyses by RT-PCR. The results are from 3 independent samples, each representing amplified cDNA originating from the macrophages from one animal. mRNA levels are presented as relative levels compared to the non-diabetic baseline group. BL = baseline, chow = chow only, 0 = HDAd-0, LDLR = HDAd-LDL. * p<0.05 vs. respective non-diabetic group (Student’s t test); # p<0.05 as labeled (one-way ANOVA).

To explore the possible molecular mechanisms underlying atherosclerotic regression, we assessed lesional macrophage gene expression using LCM samples. As shown in Fig 6, the gene expression of inflammatory cytokines as well as markers for inflammatory macrophages (TNFα, IL1β, iNOS, ICAM1 and CHOP) were reduced after switching to chow diet. Treatment with HDAd-LDLR further reduced inflammatory gene expression. In contrast, gene expression of markers for alternatively activated macrophages (considered to be anti-inflammatory), such as IL10, Arginase 1 (Arg 1) and Fizz, which were initially low in both the non-diabetic and STZ-diabetic baseline groups, increased after switching to chow diet. Treatment with HDAd-LDLR further increased the expression of these genes. In addition, the gene expression of liver X receptor alpha (LXRα) and CCR7—previously established as regression-promoting factors [27]—were low in both baseline groups, but increased after switching to chow diet, and further increased with HDAd-LDLR treatment HDAd-LDLR treatment.

Direct comparison of gene expression between the non-diabetic and STZ-diabetic mice revealed that the inflammatory cytokines and stress markers (IL1β, TNFα, ICAM1, CHOP, VEGF and iNOS) were significantly higher at baseline in the STZ-diabetic mice (p<0.05 STZ-diabetic baseline vs. non-STZ baseline). Gene expression levels of IL1β, ICAM1, CHOP and iNOS remained significantly higher after induction of regression by HDAd-LDLR treatment in the STZ-diabetic mice compared to baseline non-diabetic mice. Conversely, mRNA expression of genes associated with alternatively activated macrophages (IL10, Arg 1, Fizz) was decreased in HDAd-LDLR treated STZ-diabetic mice compared to non-diabetic mice. The changes in most of the genes noted on Fig 6 are consistent with what we previously observed in regressing plaques [27, 28] including the effects of STZ treatment [3, 8].

## Discussion

Our goal was to develop a non-surgical model of atherosclerosis regression in mice with incipient metabolic syndrome and insulin-deficient diabetes mellitus, using these as examples of the flexibility afforded by use of HDAd-LDLR treatment to study regression under differing metabolic conditions. Because many future studies of regression will require the use of mice with multiple genetic manipulations, the development of a viral-mediated cholesterol reduction system has major advantages over the use of a transplant approach or the Reversa regression model, which require either surgical expertise or extensive breeding.

Due to the prevalence of metabolic syndrome/T2DM, we first aimed to study atherosclerosis regression in a mouse model of incipient metabolic syndrome. Several studies in mice have shown that 2 weeks of HFD impairs glucose metabolism and insulin resistance in the heart [29–31]. The main fat ingredient of our HFD was palm oil, enriched in the saturated free fatty acid palmitate, which induces inflammation and apoptosis through oxidative or endoplasmic reticulum stress, and generation of ceramides and reactive oxygen species [32]. *In vivo* [33] and *in vitro* [34] studies have also shown that palmitic acid causes insulin resistance, and higher levels of palmitic acid are associated with an increased risk for T2DM in humans [35]. We hypothesized that the metabolic changes induced by palm oil-containing HFD would lead to defective atherosclerosis regression because of these adverse effects.

Using the HDAd-LDLR, we successfully lowered plasma cholesterol levels primarily in the ApoB containing lipoproteins and induced atherosclerosis regression–defined as a reduction in monocyte-derived cell content in the plaque from HFD- and LFD-fed mice. With virally-induced cholesterol reduction Oil Red O decreased and collagen content increased in the aortic sinus. However, despite incipient signs of metabolic syndrome in mice fed a palmitate-containing HFD, we did not detect differences in atherosclerosis regression compared to mice fed a LFD. There are several possible explanations for this: First, although mice displayed mild whole body insulin resistance, this was for a relatively short period on HFD (2 weeks). Our experience is that atherosclerosis regression in Reversa mice (a modified LDLr^-/-^ model) is best studied within 2–3 weeks after cholesterol reduction [8, 36] and so testing this time period seemed reasonable. Second, HDL-cholesterol was significantly elevated in mice on HFD, possibly counteracting pro-atherogenic changes like the increase in LDL-cholesterol, body weight and impaired glucose tolerance. Third, although palm oil does not contain cholesterol it can increase plasma cholesterol [37], possibly by suppressing hepatic LDLR activity and increasing LDL-cholesterol production [38]. In spite of showing higher cholesterol levels in palm oil-containing HFD-fed mice, the viral vector was so effective in lowering non-HDL cholesterol that it likely overcame differences in plasma cholesterol levels, and for that matter, mild glucose intolerance. Finally, the metabolic changes induced by HFD might not impair atherosclerosis regression.

We note that in this relatively short term regression study, we did not see visible changes when we directly viewed the aortic arch or performed Oil red O staining of the aorta. Plaque in the aortic arch is visible as a white area of non-translucency. We assume that the replacement of cholesterol and macrophage-enriched plaque by collagen led to a similar loss of transparency. En face Oil Red O staining shows the extent of the plaque, but these two-dimensional images cannot quantify the thickness of the lesions. Thus, aortic sinus sections are the most sensitive method to assess both the composition and extent of regression.

We next hypothesized that impairment of atherosclerosis regression would be more pronounced in hyperglycemic mice compared to normoglycemic mice based on our previous studies [3, 8]. Induction of regression in normoglycemic mice by HDAd-LDLR led to a dramatic reduction of both plasma cholesterol and triglycerides to levels normally found in wild type C57BL/6 mice. Switching to chow diet only also led to a decrease of plasma cholesterol to levels around 350 mg/dl. These levels are also seen in continuously chow-fed *Ldlr* ^*-/-*^ mice [39]. However, this decrease was significantly less than that in HDAd-LDLR treated mice. Accordingly, regression of atherosclerosis was most dramatic in HDAd-LDLR treated mice; lesion size, macrophage and lipid content in the lesion were significantly reduced in this group. In contrast, the two control groups showed less regression; there was no difference in the lesion size and less reduction in the macrophage and lipid content. In accord with a more stable plaque phenotype, collagen content increased only in the HDAd-LDLR treated mice. These reported changes are in agreement with other models of atherosclerosis regression after plasma cholesterol reduction. Using a Reversa mouse, Feig et al. reported a reduction of CD68+ macrophages and lipid content and increase of collagen content in atherosclerotic plaques [36]. A 50% loss of CD68+ macrophages and decrease in plaque size was reported in transplanted segments of diseased aortas into wild-type mice [27]. Studying regression 28 weeks after HDAd-LDLR treatment, Li et al. found a 54% regression in lipid-lesion area of en face aortas [14].

Turning to the effects of hyperglycemia on the response to HDAd-LDLR treatment, we assessed atherosclerosis regression in insulin-deficient diabetic mice. Compared to the normoglycemic baseline mice, cholesterol levels were significantly higher in the hyperglycemic mice, as expected [40, 41]. The higher plasma cholesterol levels likely contributed to the greater starting lesion size and CD68+ macrophage content in the STZ baseline group [22]. Nonetheless, atherosclerosis regression by HDAd-LDLR treatment had a similar overall effect compared to normoglycemic mice; the lesion size, macrophage and lipid content were significantly reduced, whereas collagen content increased. Thus, despite initially higher plasma cholesterol levels, HDAd-treatment in STZ-diabetic mice led to normalization of plasma cholesterol and induction of atherosclerosis regression.

Using the Reversa mouse model, we have previously reported that diabetes hindered plaque regression in atherosclerotic mice (based on CD68+ plaque content) and impaired favorable changes in plaque macrophage characteristics [3, 8]. Although in the current study both the normoglycemic and hyperglycemic mice were studied side by side, a direct comparison warrants caution because baseline cholesterol and atherosclerotic lesion size differed markedly between the two groups. However, calculating the percentage change in the regression groups versus their respective baseline groups showed that both reduction in lesion size and CD68+ areas were significantly less alleviated in HDAd-LDLR treated STZ-diabetic mice compared to the non-diabetic mice (51% lesion size reduction in non-diabetic mice vs. 36% in STZ-diabetic mice, and reduction of 77% CD68+ cells in non-diabetic mice vs. 57% in STZ-diabetic mice), consistent with our previous study [8]. Using hypomorphic ApoE mice, Gaudreault et al. also reported an impaired lesion size reduction with STZ-diabetes, but in contrast to our past and present results, no differences were noted in the cell composition of the atherosclerotic lesion [42]. Besides the use of different regression models, this difference might be due to different changes in lipoprotein composition (more VLDL-cholesterol reduction) and degree of cholesterol reduction, with HDAd-LDLR treatment being more effective. In concert with our data, overexpression of the VLDL receptor using a helper-dependent adenovirus resulted in aggressivelowering of triglyceride- and cholesterol-rich lipoproteins, reduced lesion size and reduced intraplaque hemorrhage in a virally-induced transgenic *Ldlr* ^*-/-*^ mouse model of type 1 diabetes [43]. Thus, the extent of cholesterol reduction and the degree of diabetes/hyperglycemia likely affects diabetic atherosclerosis regression.

We and others have reported that cell surface markers and inflammatory cytokine gene expression in plaque macrophages are increased during atherosclerotic progression [31–35] and decreased after induction of atherosclerosis regression [27, 44]. Macrophages in the inflammatory state are often referred to as M1, as opposed to those with anti-inflammatory properties [39], termed alternatively activated macrophages (M2), although acceptance of this simple classification is controversial and more likely, there is a spectrum of phenotypes between the two extremes [45]. Nonetheless, human and mouse atherosclerotic plaques contain macrophages that have either M1-like or M2-like phenotypes [46, 47], and we have consistently shown enrichment in macrophages with some M2-like features in regressing plaques of normoglycemic mice (reviewed in [44]), which would be consistent with the ability of alternatively activated macrophages to promote the remodeling of damaged tissue [48]. Using LCM to selectively remove macrophage (CD68+) foam cells from regressing and non-regressing plaques, we studied the expression pattern per cell in our model. In normoglycemic and hyperglycemic mice, mRNA levels of inflammatory genes such as TNFα, IL-1β, CHOP and ICAM-1 were reduced by cholesterol reduction, while the levels of anti-inflammatory factors such as IL-10 were increased. These effects were most pronounced when mice were treated with HDAd-LDLR. Notably, HDAd-LDLR treatment reduced the expression of iNOS, a marker for inflammatory macrophages, while the expression of markers for alternatively activated macrophages, Arg1 and Fizz [37, 38] was increased, suggesting that more alternatively activated macrophages are found in lesions during regression. CCR7 and LXRα, which play important roles in macrophage migration out of the plaque [27, 49], lipid metabolism [50], and inflammation reduction [51] were significantly increased in both normoglycemic and hyperglycemic regression mice. Of note, gene expression changes were less pronounced in HDAd-LDLR treated hyperglycemic mice compared to HDAd-LDLR treated normoglycemic mice suggesting that hyperglycemia hinders favorable changes in plaque macrophage characteristics after the reduction of total plasma cholesterol. This interpretation of our results is consistent with previous studies showing that hyperglycemia prevents conversion to alternatively activated macrophages [8] and that reduction of hyperglycemia in diabetic mice leads to fewer inflammatory circulating monocytes [3].

In summary, our studies have established a new technique to investigate atherosclerosis regression in settings of metabolic perturbation. Introduction of HDAd-LDLR led to a dramatic reduction in circulating atherogenic lipoproteins. This method obviates the need for the technically demanding use of transplants or the breeding required for regression using the Reversa model. In our studies, regression decreased the number and inflammatory phenotype of CD68+ lesional cells. Moreover, regression led to greater lesional collagen content, an indicator of repair and a more stable appearing lesion, as assessed by Sirius Red staining. Although similar benefits were seen in the hyperglycemic mice, the relative improvement in vascular lesions was significantly less than that seen in normoglycemic mice. In contrast, we did not detect differences in atherosclerosis regression in a mouse model of incipient metabolic syndrome with mild glucose intolerance. Despite the marked reduction in plasma cholesterol that is possible using high dose statins and other drugs, diabetes exerts significant vascular toxicity. In T2DM and the metabolic syndrome, factors other than hyperglycemia, including changes in the inflammatory milieu associated with obesity, are likely to prevent normal atherosclerosis regression, and the versatile approach afforded by HDAd-LDLR treatment will make possible insightful investigations of these and other dysmetabolic settings.

## Supporting Information

S1 Table Diet content. (TIF)

S2 Table Primers and probes for RT-qPCR of CD68+ cells. (TIF)

S1 Fig (A) Quantification of Oil Red O positive lipids and (B) collagen-positive area in the aortic sinus of non-diabetic baseline and regression mice. Bar = 100μm. **: p<0.01, ***: p<0.001.
(TIF)

S2 Fig (A) Quantification of Oil Red O positive lipids and (B) collagen-positive area in the aortic sinus of STZ-diabetic baseline and regression mice. Bar = 100μm. *: p<0.05, **: p<0.01, ***: p<0.001.
(TIF)
