# Supplementary figures and images for: Effects of High Fat Feeding and Diabetes on Regression of Atherosclerosis Induced by Low-Density Lipoprotein Receptor Gene Therapy in LDL Receptor-Deficient Mice
Source: PLoS One. 2015 Jun 5;10(6):e0128996. doi: 10.1371/journal.pone.0128996 (PMC4457481; doi:10.1371/journal.pone.0128996)

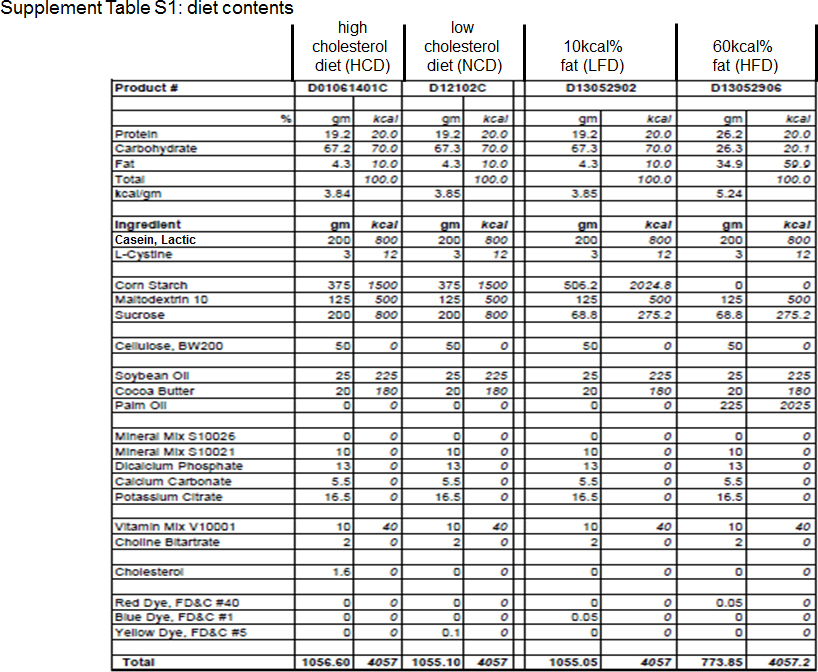

Supplement: S1 Table — (TIF) [file pone.0128996.s001.tif]

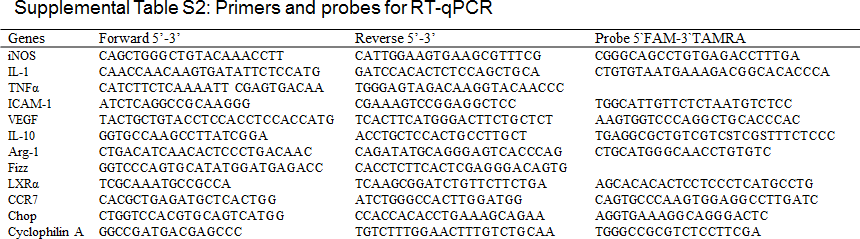

Supplement: S2 Table — (TIF) [file pone.0128996.s002.tif]

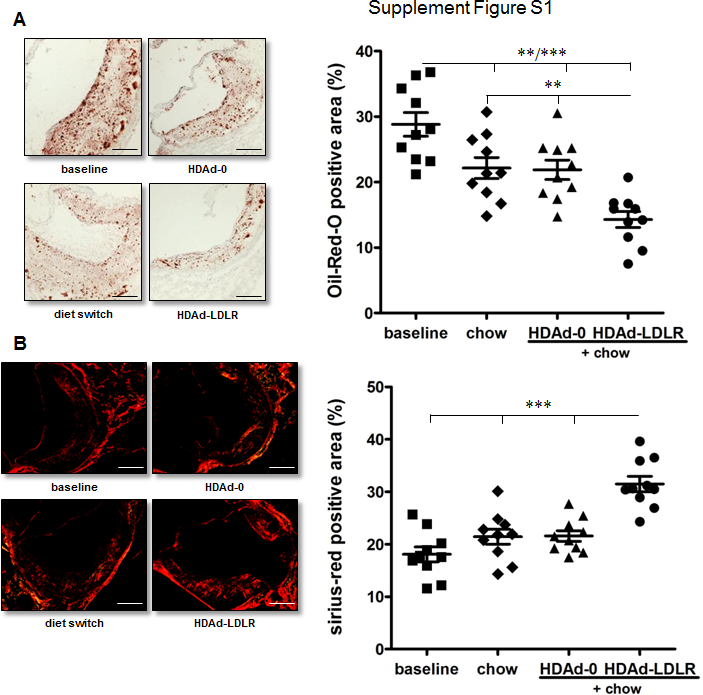

Supplement: S1 Fig — Bar = 100μm. **: p<0.01, ***: p<0.001. (TIF) [file pone.0128996.s003.tif]

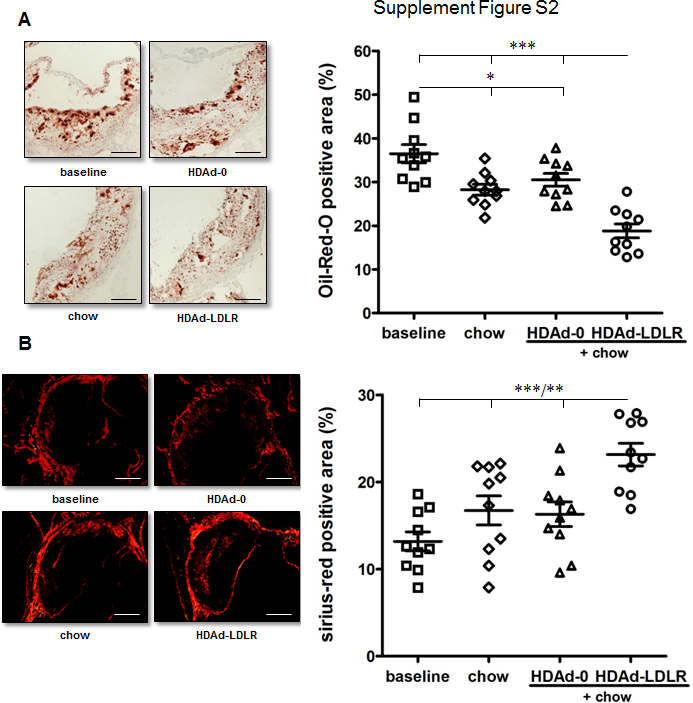

Supplement: S2 Fig — Bar = 100μm. *: p<0.05, **: p<0.01, ***: p<0.001. (TIF) [file pone.0128996.s004.tif]
